# Infant-Caregiver Experiences Alter Telomere Length in the Brain

**DOI:** 10.1371/journal.pone.0101437

**Published:** 2014-07-01

**Authors:** Arun Asok, Kristin Bernard, Jeffrey B. Rosen, Mary Dozier, Tania L. Roth

**Affiliations:** 1 University of Delaware, Department of Psychological and Brain Sciences, Newark, DE, United States of America; 2 Stony Brook University, Department of Psychology, Stony Brook, NY, United States of America; Radboud University, Netherlands

## Abstract

Following adverse childhood experiences, high quality maternal care can protect against accelerated telomere shortening in peripheral cells. It is less clear, however, how telomere length in the brain is influenced by early caregiving experiences. Using rats, we investigated if quality of care (i.e., aversive or nurturing care outside of the homecage) during the first seven days of postnatal (PN) life affected telomere length in the adult brain (PN90) of male and female rats. At PN90, we found that nurturing care outside of the homecage was associated with longer telomeres in the medial prefrontal cortex relative to nurturing care inside the homecage (i.e., normal maternal care) and aversive care outside of the homecage. Further, pups exposed to aversive care outside of the homecage demonstrated longer telomeres in the amygdala relative to pups exposed to nurturing care inside the homecage. These effects were specific to females. No differences in telomere length between caregiving conditions were observed in the ventral hippocampus. Thus, positive and negative early-life experiences result in long-term, sex-specific changes of telomeres in the brain.

## Introduction

Maltreatment and chronic stress in childhood are associated with numerous impairments to future physical and mental health, such as anxiety and depression [1], [2]. There is an increasing urgency to discover biomarkers that can discriminate between increased risk susceptibility and resiliency following early-life stress. Telomere length in peripheral cells has emerged as a biomarker of the impact of early-life and chronic stress [3]. Telomeres are long stretches of TTAGGG nucleotide repeats that cap the ends of DNA [4]. Telomere length plays a role in cellular aging with both biological and psychological environmental factors influencing the rate of their attrition in peripheral cells [3], [5], [6]. However, the pattern of changes to telomere length differs across gender, cells, organs, and disease-states [7], [8], [9], [10], [11], [12].

Individuals exposed to chronic psychological stress during childhood (e.g., prolonged institutional care, maltreatment, exposure to violence) display accelerated telomere shortening in peripheral cells (i.e., buccal cells and leukocytes; [13], [14], [15], [16]). We recently showed that high quality responsive maternal care appears to protect telomeres from accelerated shortening in 4- to 6-year-old children living under chronically stressful conditions [16]. Although there is evidence that changes in telomere length in peripheral cells and organs are associated with stress, very little is known with regard to how telomeres change in the brain following stress – particularly early-life stress. Rodent models of early-life stress allow for the examination of stress-related mechanistic changes to telomere length in the brain. Only a few studies to date have investigated the effects of stress on telomeres in rodents [17], [18], [19].

The present study used a model of early aversive and nurturing caregiving in rats [20], [21], [22] to examine whether variations in the caregiving environment for 30 minutes a day during the first week of postnatal life influenced telomere length in different brain regions - the medial prefrontal cortex (mPFC), amygdala, and the ventral hippocampus (vHPC). The end of the first postnatal week in the rat is considered the neurodevelopmental equivalent of a near-term human infant, and these brain regions were selected because childhood maltreatment and other types of early adversity have been shown to alter function of the prefrontal cortex, amygdala, and hippocampus [23], [24], [25],[26]_ENREF_12.

## Materials and Methods

### Ethics statement

All procedures were approved by the University of Delaware's Institutional Animal Care and Use Committee.

### Subjects

Male and female Long-Evans pups obtained from our breeding colony were used. Analyses for the present study included animals from 16 litters used in several recent DNA methylation studies [21], [22]. Mothers were housed in opaque polypropylene cages with wood shavings and kept at constant temperature (25°C) under a 12-hour light/dark cycle (lights on at 6:00 AM), with *ad libitum* access to food and water. Day of parturition was termed 0 days of age, and litters were culled to 6 males and 6 females on postnatal (PN) day 1. All behavioral manipulations and observations occurred during the light cycle between approximately 8 a.m. – 5 p.m.

### Caregiver manipulations

A within-litter design was used for all caregiver manipulations performed between PN1-PN7. On PN1, male and female pups in a litter were randomly divided into one of three caregiving conditions: (1) foster care (nurturing); (2) maltreatment (aversive); or, (3) normal maternal care (nurturing). Up to 2 males and 2 females were distributed across each condition. For the foster care condition, pups were removed from the homecage and placed with another lactating dam for 30 minutes a day. To promote nurturing care, the foster care dam was given 1 hour to habituate to her experimental chamber and given sufficient nesting material. The procedure for the maltreated pups was identical except that another lactating dam was given insufficient nesting material and time to habituate to her experimental chamber. Remaining littermate controls were marked and weighed, but immediately returned to the biological mother (to serve as normal maternal care controls).

Reduced nesting material and insufficient time to habituate to the experimental chamber have been shown to induce overt maltreatment-like behaviors (e.g., dragging, stepping-on, rough handling) in lactating dams [20], [21], [22]. This model is a variation on that introduced by the Baram Laboratory [27], [28] and used by the Sullivan Laboratory [29], [30], which capitalizes on the ability of resource deprivation (nesting material) to produce aberrant, often maltreatment-like, behaviors of dams, even within the homecage. The brief (30 minutes/day) and repeated (7 days) nature of the exposures capitalizes on the ability of pups to rapidly learn about their environments during a period of brain development sensitive to maternal influences [31], [32].

All exposure chambers were maintained at 27–30 °C. Time of day for exposure sessions across the 7 day regimen was varied to make the time for exposures unpredictable. From PN8, litters remained undisturbed until weaning (PN21-23), when mothers were removed from the homecage. At PN30, subjects from the same experimental condition and same sex were then housed in groups of 2–3. Thus, aside from the 7-day 30 minute exposures and weaning, subjects did not receive any additional manipulations and their experiences (food, cage changes, etc.) were identical.

### Analysis of caregiver and pup behavior

Using methods previously reported [20], [21], [22], caregiving behavior during the 30-minute exposure sessions was scored by 2 trained experimenters (either from live observations or video recordings). Each 30-minute session was separated into 5-minute time bins in which pup-caregiver interactions were quantitatively assessed. Nurturing and adverse caregiving behaviors (stepping on, dropping, dragging, avoiding, roughly handling, licking/grooming, hovering/nursing) were scored by tallying whether a type of behavior occurred or did not occur within each 5-minute time bin. Behavior scores were averaged across all 7 postnatal days for each condition. We also measured audible and ultrasonic (recorded at 40 kHz) vocalizations of pups in response to caregiving behaviors during the 30-minute sessions for each postnatal day (Batbox IIID, NHBS Ltd.,UK). Ultrasonic vocalizations were scored by simply tallying whether a vocalization, regardless of its duration, occurred or did not occur within each minute time bin during a thirty minute session. Vocalizations were then averaged across the seven exposure days and sessions (as we did for caregiving behaviors). Audible vocalizations were scored and summarized in the same manner. Due to the design of the chambers and number of pups in the chambers at a time (up to 4), we were not able to attribute specific types of caregiving behaviors or vocalizations to individual male or female pups during the exposure sessions.

### Tissue collection

We chose to collect tissue at PN90 given that our previous studies using this same animal model and cohort have shown that long-term changes in DNA methylation, alterations in the levels of epigenetic regulators, and changes in gene expression are present at this age within the same 3 brain regions that are the focus of this study [20], [21], [22], [33]. At PN90, animals were removed from their home cages and immediately sacrificed. Brains were removed, sliced using a 1 mm brain matrix, flash frozen on untreated microscope slides with 2-methylbutane on dry ice, and placed in a −80 freezer until later processing. Regions corresponding to the medial prefrontal cortex (mPFC), amygdala (basolateral, lateral, central amygdala nuclei combined), and ventral hippocampus (vHPC) were removed freehand with a scalpel using the Paxinos and Watson stereotaxic atlas as a guide [34]. DNA was extracted via the manufacturer's instructions (Qiagen AllPrep DNA/RNA kit, Valencia, CA) and stored at −80C until use.

### Relative telomere length assay

DNA was quantified, assessed for purity, and diluted to 10 ng/uL via Nanodrop spectrophotometry following procedures used previously [16]. Quantitative real-time PCR (qRT-PCR) was conducted with primer sets targeting telomeres (T; Forward_TEL_:5′-CGGTTTGTTTGGGTTTGGGTTTGGGTTTGGGTTTGGGTT-3′ and Reverse_TEL_:5′-GGCTTGCCTTACCCTTACCCTTACCCTTACCCTTACCCT-3′) and the single copy control gene (S), acidic ribosomal phosphoprotein 36B4 (Forward_36B4_: 5′- ACTGGTCTAGGACCCGAGAAG-3′ and Reverse_36B4_: 5′-TCAATGGTGCCTCTGGAGATT-3′) [35]. Each PCR well contained a final concentration of 1× Power Sybr Green Master Mix (Life Technologies, Grand Island, NY), 100 nM Forward Primer, 100 nM Reverse Primer, and 20 ng of sample DNA, and the experimenter was always blind to a sample's group identification when loading the PCR plate. For each sample, the telomere and single copy gene qRT-PCR assays were carried out in triplicate in the same well position on different 96-well plates. Male and female samples were run on different 96-well PCR plates that contained a representative sample of each caregiving condition. All samples from a particular brain region for a particular sex (e.g., all female mPFC samples) were analyzed on a single PCR plate to allow for comparisons across the three caregiving conditions. Given that plates did not contain a standard, we did not statistically contrast samples between different PCR plates. All samples within a plate were assayed in triplicate and any sample replicate that deviated ± 1 cycle threshold (Ct) beyond the triplicate average was excluded from the final Ct average of each sample. In these cases (< 5.4%), the remaining two replicates were used for the sample average. Relative telomere length was calculated as a ratio of telomeres to single copy gene and transformed into a fold change relative to normal maternal care for the particular sex in a particular region. Relative telomere length was first calculated by the formula T/S = (2^ΔCt tel^)/(2^ΔCt 36B4^) and then transformed to a proportionate score against normal maternal care for that particular sex in a particular region (Fold Change_sample_  =  (T/S_sample_/T/S _Avg. NMC_). Scores were transformed into a fold change relative to normal maternal care controls to allow for visual discrimination of the change in magnitude relative to controls across all brain regions and sexes. This transform is similar to the approach we and others have applied for epigenetic analyses [20], [22].

### Data analytic approach

Because qRT-PCR plates contained only one sex (i.e., only males or only females) and samples from one brain region, statistical analyses were conducted independently for each sex and brain region. For each plate (which only contained a single sex, but all caregiving conditions), any sample that was ±1.96 std. deviations above or below the group mean was excluded as an outlier (mPFC: n_female_ =  2 (1 maltreatment and 1 normal care), n_male_  =  2 (1 maltreatment and 1 normal care), Amygdala: n_female_  =  2 (2 foster care), n_male_  =  3 (1 foster care, 1 maltreatment, and 1 normal care), vHPC: n_female_  =  6 (2 foster care, 2 maltreatment, and 2 normal care), n_male_  =  3 (1 foster care, 1 maltreatment, and 1 normal care). The final number of rats after exclusion of outliers for each sex were as follows: mPFC: n_female_  =  29 (foster care  =  6, maltreatment  =  11, normal care  =  12), n_male_  =  29 (foster care  =  10, maltreatment  =  9, normal care  =  10); Amygdala: n_female_  =  24 (foster care  =  11, maltreatment  =  6, normal care  =  7), n_male_  =  15 (foster care  =  5, maltreatment  =  7, normal care  =  5); and, vHPC: n_female_  =  24 (foster care  =  8, maltreatment  =  8, normal care  =  8), n_male_  =  44 (foster care  =  16, maltreatment  =  9, normal care  =  19). Separate one-way ANOVAs were conducted to test for main effects of caregiving condition on telomere length in each brain region. To follow-up on significant main effects, stringent pairwise comparisons with a Bonferroni correction were conducted to identify specific differences between caregiving conditions.

## Results

### Caregiver and pup behavior

**Figure 1:**
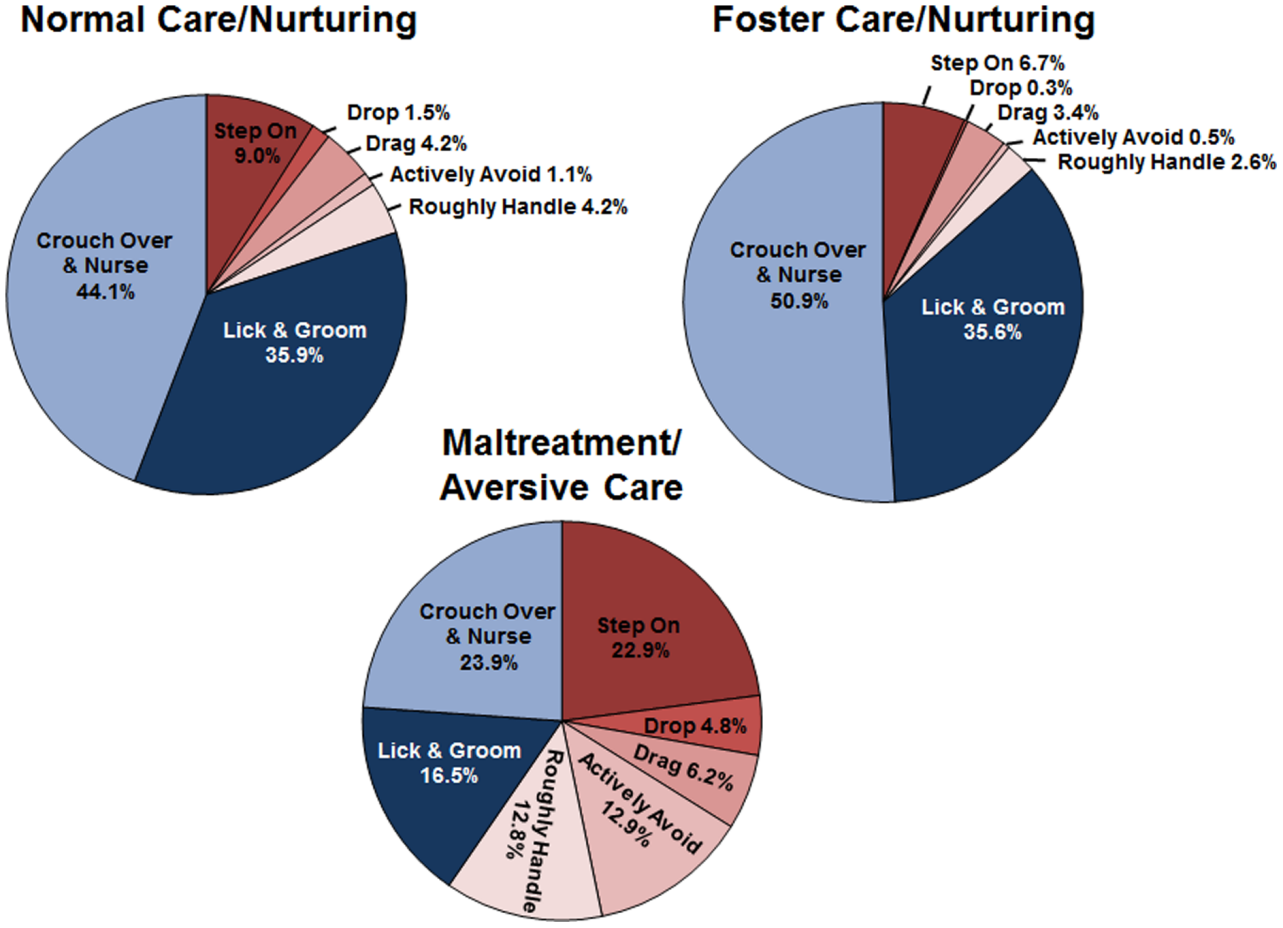
Pups experienced different caregiving environments. During the 30-minute exposure sessions, pups in both the normal and foster care conditions received predominately nurturing behaviors consisting of pup licking, grooming, crouching over, and nursing. Pups in the maltreatment condition instead experienced far greater amounts of adverse caregiving behaviors including dragging, dropping, stepping-on, and less nurturing behaviors.

As previously reported for this cohort [21], [22] and others [20], rat pups exposed to an aversive caregiving environment displayed more audible and ultrasonic vocalizations, *p*'*s* < .05, than pups exposed to nurturing caregiving environments either outside of or within the homecage (Table 1). Further, maltreated pups experienced more frequent aversive caregiving behaviors (e.g., stepping on, rough handling), *p*'*s* < .05, and less frequent nurturing caregiving behaviors (e.g., nursing and licking/grooming), *p*'*s* < .05, than littermate controls (Figure 1 and Table 1). There were no differences in pup vocalizations or caregiving behaviors between the foster and normal care conditions.

**Table 1:** Statistical contrasts between observed behaviors across caregiving conditions.

	Normal Care vs. Maltreatment	Foster Care vs. Maltreatment	Normal Care vs. Foster Care
**Adverse Caregiving Behaviors**			
Step On *F*(2,32) = 23.02, *p* < .001	***p*** ** < .001**	***p*** ** < .001**	N.S.
Drop *F*(2,32) = 7.52, *p* < .001	N.S.	***p*** ** < .01**	N.S.
Drag F(2,32) = 1.81, *p* > .05	N.T.	N.T.	N.T.
Avoid F(2,32) = 7.88, *p* < .01	***p*** ** < .05**	***p*** ** < .01**	N.S.
Roughly Handle F(2,32) = 19.39, *p* < .001	***p*** ** < .01**	***p*** ** < .001**	N.S.
**Nurturing Caregiving Behaviors**			
Lick/Groom F(2,32) = 7.83, *p* < .01	***p*** ** < .05**	***p*** ** < .01**	N.S.
Crouch Over/Nurse F(2,32) = 12.89, *p* < .001	***p*** ** < .05**	***p*** ** < .001**	N.S.
**Pup Vocalizations**			
Audible F(2, 15) = 6.26, *p* < .05	***p*** ** < .05**	***p*** ** < .05**	N.S.
Ultrasonic F(2, 12) = 20.84, *p* < .001	***p*** ** < .01**	***p*** ** < .001**	N.S.

	Normal Care	Foster Care	Maltreatment
Audible (% Time Vocalizing)	29.33%	25.30%	45.2%
Ultrasonic (% Time Vocalizing)	57.98%	46.5%	85.6%

*Bold text indicates statistically significant differences.

*N.T. indicates no post-hoc test was conducted because no main effect was detected.

*N.S. indicates not significant.

### Relative telomere length

**Figure 2:**
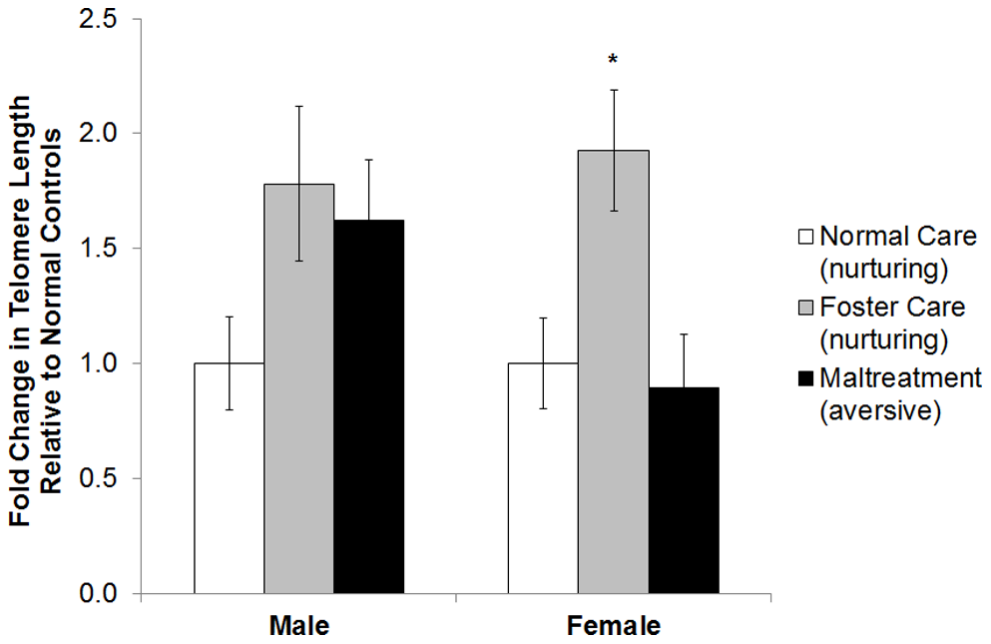
Nurturing care is associated with longer telomeres in the adult female mPFC. Females exposed to nurturing care outside the homecage between PN1-7 exhibited longer telomeres in the mPFC at PN90 relative to littermates that received either maltreatment or normal care, *p < .05. Males did not statistically differ. Error Bars are ± S.E.M.

In the mPFC, there was a significant effect of caregiving condition on telomere length in females *F*(2, 26)  =  4.52, *p* < .05, but not males *F*(2, 26)  =  2.31 *p* > .05. Post-hoc analyses showed that foster care females had significantly longer telomeres relative to normal care and maltreated females (*p*'*s* < .05; Figure 2). Normal care and maltreated females did not differ in mPFC telomere length.

**Figure 3:**
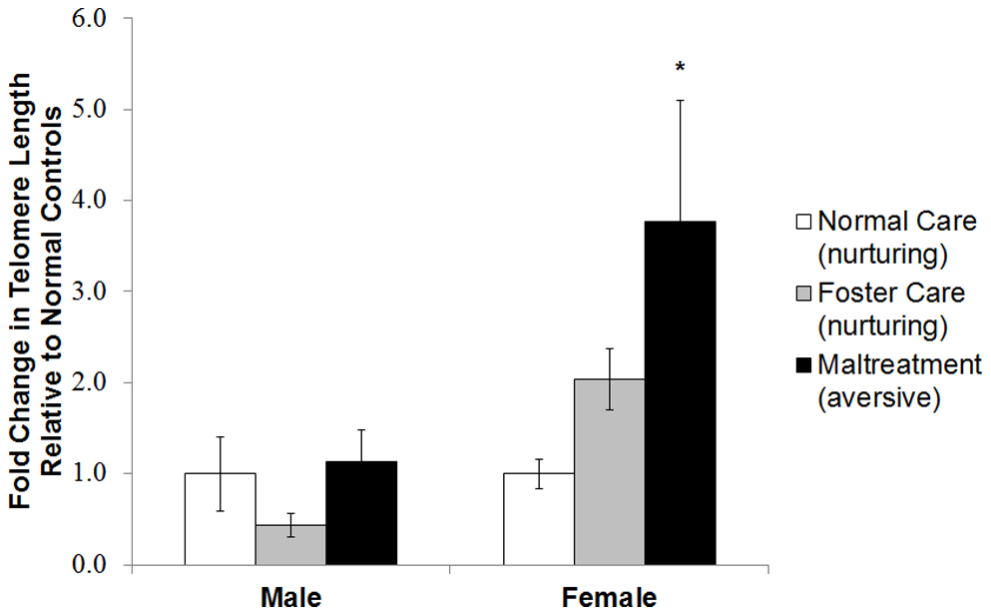
Aversive care is associated with longer telomeres in the adult female amygdala. Females exposed to maltreatment between PN1-7 exhibited longer telomeres in the amygdala at PN90 than littermates exposed to normal care, *p < .05, but did not significantly differ from nurturing care, *p* > .05. Males did not statistically differ. Error Bars are ± S.E.M.

**Figure 4:**
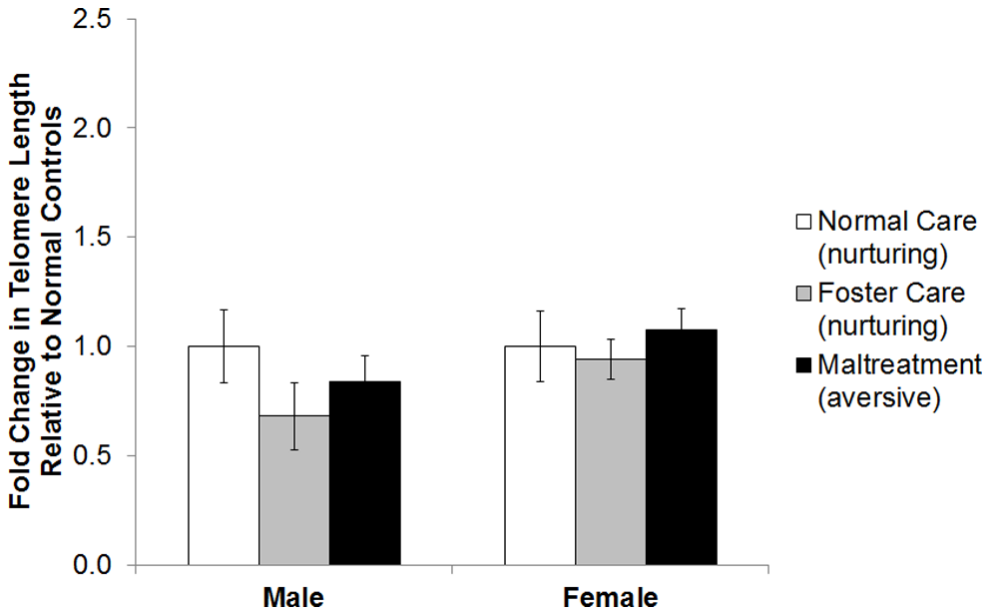
Caregiving behavior is not associated with telomere length in the vHPC. No significant effect of early-life condition on telomere length was observed in the ventral hippocampus for males and females at PN90.

In the amygdala, there was a significant effect of caregiving condition on telomere length in females *F*(2, 22)  =  4.54, *p* < .05, but not males *F*(2, 14)  =  1.25, *p* > .05. Post-hoc analyses showed that maltreated females had longer telomeres relative to normal maternal care females, *p* < .05, but not compared to foster care females, *p* > .05 (Figure 3). Finally, in the ventral hippocampus, there was no effect of caregiving condition on telomere length for females *F*(2, 21)  =  .29, *p* > .05, or for males *F*(2, 41)  =  .98, *p* > .05 (Figure 4).

## Discussion

This study demonstrates for the first time that exposure to nurturing or aversive caregiving environments outside the homecage in infancy result in long-term changes in telomere length within the brain. These changes were found in different brain regions of adult female, but not male, rats. Remarkably, only 30 minutes of nurturing foster or aversive care per day during the first 7 days of life resulted in these long-term differences in telomere length.

First, we found that female rats exposed to nurturing foster care outside of the homecage exhibited longer telomeres in the mPFC than females exposed to maltreatment or normal care. Both foster care and normal care female rats experienced significantly more positive caregiving behaviors (i.e., licking/grooming and crouch over nursing) than maltreated females, but did not differ from each other - reducing the likelihood that this effect is due to any enhancement in levels of nurturing care by handling [36]. However, it should be noted that foster care pups (both male and female) did experience nurturing care from multiple dams (i.e., inside the homecage and outside the homecage) over the first 7 postnatal days. Thus, although only females exhibited longer telomeres, this factor (i.e., exposure to multiple nurturing dams) may contribute to our results. Our second major finding is that maltreated females had longer telomeres in the amygdala than females who experienced normal maternal care. Maltreated females received significantly more aversive caregiving behaviors (i.e., stepping-on, avoidance, and roughly handling) than both normal care and foster care females. Together, these data indicate that central nervous system (CNS) telomere length in females is especially sensitive to variations in caregiving environments, and future studies will be necessary to determine which specific factors within each condition are responsible for the observed effects.

Our findings are consistent with previous studies demonstrating that early-life experiences (1) lead to molecular changes in the mPFC and amygdala, and (2) may differentially influence these regions in males versus females. Changes in the caregiving environment are known to change the developmental trajectory of prefrontal-amygdala connectivity [37] and induce sex-specific molecular, structural, and functional changes in the mPFC and amygdala [38], [39], [40], [41]. For example, maternal separation is known to alter dendritic growth and spine density in the mPFC in a sex-specific manner [42]. Additionally, maternal separation has been shown to reduce the number of astrocytes in the prefrontal cortex in male and female rats – suggesting the effects of early-life stress can extend to various cell types [43]. Communal nesting, a form of an enriched early-life environment where pups have high levels of both maternal and peer interactions because of cohabitation of multiple mothers and their offspring, has positive effects on brain and behavioral systems, including increased BDNF levels in the frontal cortex [44]. Further, female mice are more sensitive to the beneficial effects of communal nesting on adult depression-like behavior than male mice [45]. It is possible that the enriched social environment that our foster care females experienced (i.e. nurturing care from multiple caregivers) slowed the normal rate of telomere loss [46] and associated structural changes that could occur with development within the PFC [47], [48].

Early-life stress (i.e., maternal separation) has been shown to enhance aversive amygdala-dependent learning and memory in female, but not male rats [49], [50]. Male and female rats naturally exhibit increased dendritic branching in the basolateral amygdala with development (between PN35-PN90), and stressful experiences (chronic and acute) have been shown to further increase this dendritic remodeling (i.e. hypertrophy) within the amygdala [47], [51]. The impact of early-life stress in the amygdala also extends to changes in glial populations – providing further support for the notion that changes in telomere length may be reflective of changes in a variety of cells throughout the brain [43], [52]. Reduced cellular proliferation has been suggested to account for some observations of longer telomere length in the brain [53], and early-life stress may decrease cellular proliferation during development in the amygdala [54], [55], [56]. It is possible that our observation of longer telomeres in the amygdala of maltreated females could reflect environmentally-driven changes in a variety of cell-types and overall changes in cellular proliferation [57].

Beery et al. showed that young adult rats exposed to a variety of stressors for 12 weeks (e.g., swim stress, fox urine, restraint) demonstrated increased telomerase (i.e., the enzyme that replenishes telomere repeats) activity in leukocytes and alterations in corticosterone secretion [17]. Further, a recent study showed that overexpression of telomerase reverse transcriptase (TERT), a catalytic subunit of telomerase, in the hippocampus of mice increased neurogenesis, reduced depressive-like behavior in a forced swim task, and attenuated the deleterious effects of three weeks of chronic mild stress (e.g., restraint stress, forced-swim, food/water deprivation [19]). Thus, our observation of longer telomeres in the amygdala and mPFC might also reflect environmentally-driven changes in telomerase activity.

We did not observe an effect of caregiving condition on telomere length in the ventral hippocampus, which differs from findings reported in a previous study [18]. Botha et al. (2012) examined the effect of prolonged maternal separation in infancy (i.e., 3 hours per day PN2-14) on telomere length in adult male rats. At PN65, maternally-separated male rats had longer telomeres in cells of the ventral hippocampus, an area of high proliferative capacity, than rats that were not maternally separated. In contrast, maternal separation did not affect telomere length in the medial prefrontal cortex [18]. Although we did not observe an effect of aversive care on telomere length in the ventral hippocampus of males at PN90, our study differed from that of Botha et al. in a number of substantial ways including: the length and type of maternal manipulation (daily 30-minute sessions outside of the home cage from PN1-7 vs. daily 3-hr. separations from PN2-14), type of tissue extracted (bilateral vs. unilateral), and, the age at which tissue samples were taken (PN90 vs. PN65).

Telomere length in the peripheral nervous system (PNS) is known to be influenced by many factors including: oxidative stress, metabolic stress, psychological factors, and exercise [3], [6], [58], [59], [60], [61]. It is unknown whether telomere changes in the PNS parallel those observed in the brain (including the natural shortening that occurs with age [7], [62]). Our data, and those from the Botha et al. study [18], indicate that particular forms of early-life experiences may be associated with longer, rather than shorter, telomeres in different parts of the brain. Interestingly, although shorter telomere length has been reported in the periphery of Alzheimer's patients, longer telomere length has been found in hippocampal cells – speculatively suggesting an inverse relationship between PNS and CNS telomere length under conditions of added stress in specific brain regions [62]. However, more research is needed to better understand the relationship between PNS and CNS telomere length throughout the lifespan and following stressful experiences [63].

Given our findings that telomeres in the CNS are sensitive to early-life environments, future studies should examine potential mechanisms involved in regulating CNS telomere length. Changes in telomere length within the brain might be mediated by telomerase function in specific cell types (e.g., neurons vs. different glial cells; [57]). Further, the chromatin structure of subtelomeric regions can undergo epigenetic changes (e.g., methylation) similar to those of genes associated with plasticity [8], [64]. Subunits of telomerase can also directly influence the transcription of genes [8], [65], [66]. During development of the nervous system, DNA damage (e.g., oxidative stress [67]) may recruit telomeric enzymes (i.e., telomerase and TRF2) to affect chromatin structure [8] – providing support for an important association between telomeres and early-life stress. In fact, both aversive and nurturing care outside of the homecage have been shown to alter chromatin structure and mRNA levels of epigenetic regulators in the mPFC and amygdala [21], [22], [33].

These properties of telomeres and telomerase, coupled with the findings of the present study, offer a myriad of potential directions for future research. For example, how do telomere dynamics (i.e., telomere length and telomerase) differ across cell types (i.e., neurons and glia) and across brain regions (e.g., mPFC, amygdala, etc.) following behavioral manipulations? How can alterations to telomerase activity in brain alter the impact of stress on telomeres and other cellular processes? What type of relationship exists between stress-related hormone and neuropeptide changes (e.gs glucocorticoids, corticotropin releasing hormone) and telomere dynamics? How do epigenetic alterations to subtelomeric chromatin structure in response to early-caregiving and adult stress relate to future behavior? Given that the structure of telomeres is highly conserved between humans and rats, investigation of CNS telomere dynamics offers a novel approach for understanding the cellular effects of early-life stress in relation to future physical and mental health.
